# Near-infrared fluorescence-assisted thoracoscopic diverticulectomy of esophageal diverticulum: a case report

**DOI:** 10.1186/s13019-023-02142-3

**Published:** 2023-03-10

**Authors:** Hao Xu, Tian Guan, Rongrong Fan, Fan Yang, Yun Li, Jian Zhou

**Affiliations:** Department of Thoracic Surgery, Peking University People’s Hospital, Beijing, 100044 China

**Keywords:** Esophageal diverticulum, Diverticulectomy, Indocyanine green, Near-infrared fluorescence, Case report

## Abstract

**Background:**

Esophageal diverticulum is a rare condition that requires treatment only when symptoms are present. Surgery has been considered to be the only curative option for symptomatic cases. The most popular procedure is diverticulectomy. Clear and intact exposure of the diverticulum’s neck is the basis for safe and effective diverticulectomy.

**Case presentation:**

We herein report a case of a 57 year-old woman with an epiphrenic diverticulum. VATS diverticulectomy was scheduled. To better identify the diverticulum neck, we injected indocyanine green (ICG) into the diverticulum through the endoscopic channel, and the diverticulum wall and neck were clearly visible under near-infrared (NIR) fluorescence. With the help of this method, diverticulectomy was successfully performed.

**Conclusion:**

This case shows that NIR fluorescence with ICG is safe, simple and reliable and can be used for diverticulectomy.

## Background

Esophageal diverticulum is a rare benign abnormality defined as a protrusion from the esophagus. The majority of patients are asymptomatic, and only symptomatic patients require treatment [1]. The last decades have witnessed a spurt in minimally invasive surgery (MIS), and there is a trend to use MIS, including video-assisted thoracic surgery (VATS) and laparoscopic surgery, to treat esophageal diverticula [2]. Regardless of the methods used, clear and intact exposure of the diverticulum neck is the basis of a safe and efficient resection of the diverticulum [3]. Even with the assistance of endoscopy, it is sometimes difficult to identify the diverticulum neck, especially when the esophageal diverticulum is tightly adhered to the surrounding tissue. Here, we describe a case in which we utilized indocyanine green (ICG) and near-infrared (NIR) fluorescence to identify the neck of an epiphrenic diverticulum. Consent was obtained from the patient.

## Case presentation

**Fig. 1:**
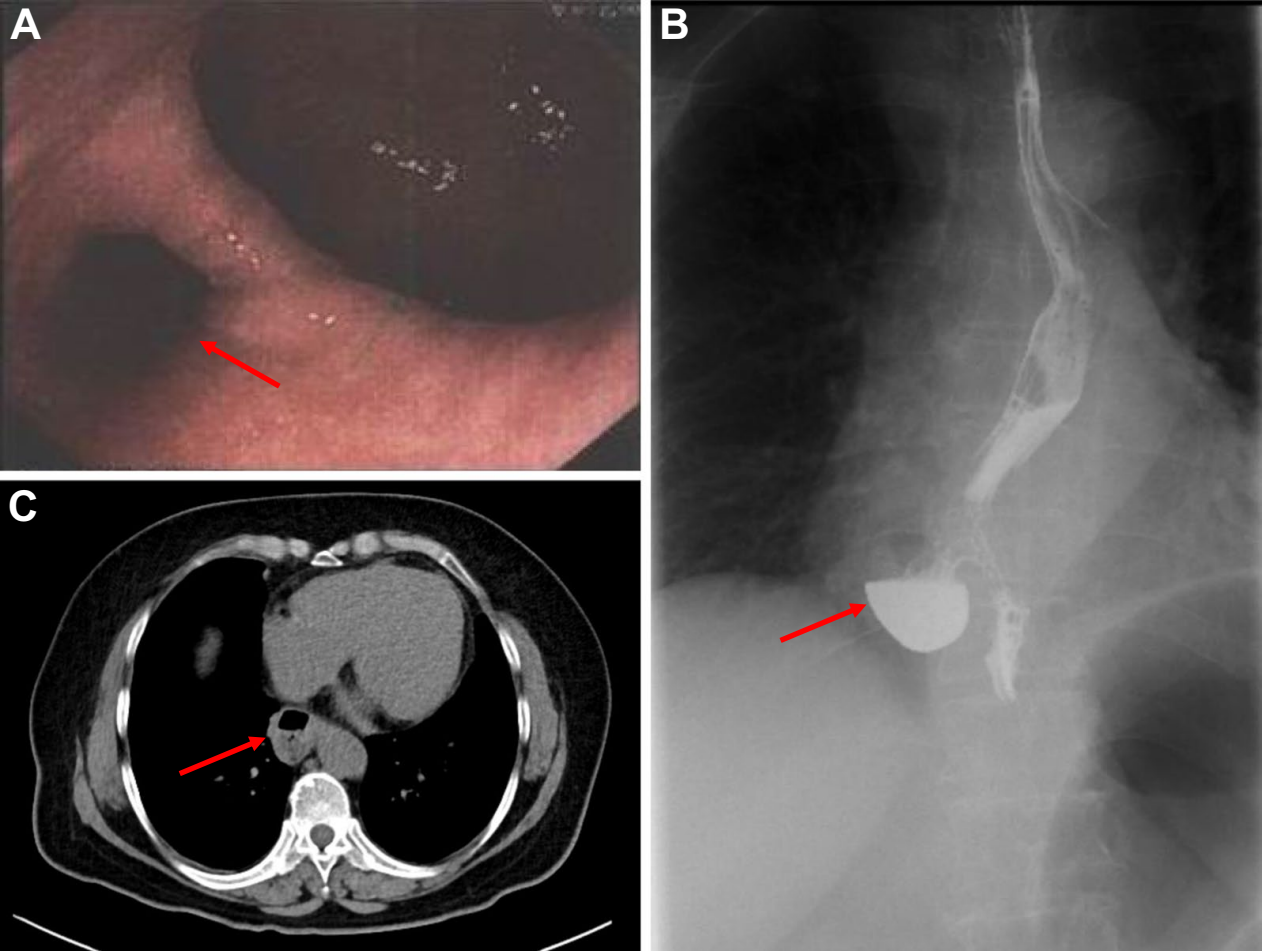
**A** Upper gastrointestinal endoscopy showed the diverticulum 30 cm from the incisor line. **B** Upper gastrointestinal contrast examination revealed a diverticulum on the right wall, and barium remained in it. **C** Chest enhanced computed tomography revealed an epiphrenic diverticulum with a diameter of 35 mm

The patient was a 57 year-old woman who presented with the chief complaint of retrosternal foreign body sensation for 3 months. She visited a hospital near her home, and upper gastrointestinal endoscopy revealed a diverticulum 30 cm from the incisor line (Fig. 1A). She had a medical history of hypertension for 25 years. Upon admission, physical examination, chest X-ray study, lung function test and laboratory test were all normal. An upper gastrointestinal contrast examination revealed a diverticulum on the right wall, and barium remained in the diverticulum (Fig. 1B). Chest enhanced computed tomography revealed an epiphrenic diverticulum with a diameter of 35 mm (Fig. 1C).

**Fig. 2:**
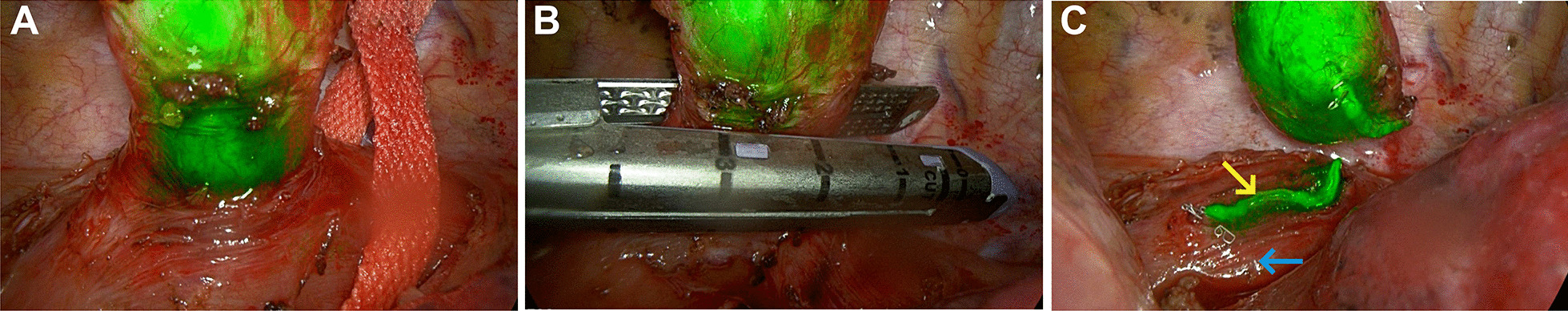
**A** The diverticulum neck stained with ICG is visualized under NIR fluorescence. **B** The neck was resected with an endoscopic purple stapler exactly parallel to the longitudinal axis of the esophagus with the assistance of ICG. **C** The diverticulum was excised, and ICG-stained diverticulum walls and neck were seen. The mucosa of the esophagus is stained (yellow arrow), and the normal muscularis is not (blue arrow)

VATS diverticulectomy was scheduled. The patient was placed in the left lateral position and under general anesthesia using double lumen intubation to collapse the right lung. The operation port and observation port were made in the fifth and seventh intercostal spaces, respectively. We first identified the esophageal diverticulum in the posterior mediastinum and incised the overlying pleura. We found that the diverticulum was slightly more adherent to the surrounding lung tissue, and the diverticulum wall was carefully dissected. We further dissected the neck region of the diverticulum with blunt and sharp dissection. With the aid of an endoscope, the diverticulum neck was identified bit by bit with the help of the endoscopic light. To better identify the diverticulum neck, we inserted the lens of the endoscope into the diverticulum and instilled approximately 30 ml ICG (2.5 mg/ml) into the diverticulum through the endoscopic channel. The wall and neck of the diverticulum were visualized immediately under NIR fluorescence, whereas the normal esophagus could not be seen due to the occlusion of the muscular layer (Fig. 2A). To avoid causing esophageal stenosis, we used the endoscope as a bougie. Then, the neck was resected with an endoscopic purple stapler (EndoGIA) exactly parallel to the longitudinal axis of the esophagus with the assistance of ICG (Fig. 2B). The overlying muscle layers were reapproximated over the stapler suturing with V-lock suture (Covidien, Inc.). An air-leak test was performed to rule out early leakage. A chest tube was placed, and the specimen was extracted. The operation time was 122 min, and the intraoperative blood loss was 40 ml.

The postoperative course was uneventful. On postoperative day 3, the patients started oral intake, and the thoracic drain was removed. The patient was discharged on day 5. During the follow-up six months after the operation, there was no recurrence of retrosternal foreign body sensation.

## Discussion and conclusions

Surgery has been considered to be the only curative option for symptomatic diverticula [1]. Traditionally, thoracotomy and laparotomy are standard procedures for diverticula. With the advancement of technology, laparoscopy and thoracoscopy are increasingly used for treatment [2]. The most popular procedure is diverticulectomy. There are controversies regarding the application of myotomy and fundoplication [4]. In short, the method of treatment selected needs to be determined according to the specific situation of the patient. In the present case, the patient had no signs of esophageal motility diseases, and myotomy and antireflux procedures were not performed.

Full exposure of the neck is the basis for satisfactory resection of the diverticulum [3]. Inaccurate identification of the diverticulum neck may lead to incomplete resection of the diverticulum, resulting in the transformation of a large diverticulum into a small diverticulum, and may also lead to intraoperative iatrogenic esophageal rupture, which may increase the possibility of postoperative esophageal stenosis or leakage. We usually identify the neck under endoscopic intraluminal vision and the endoscopic light shining through the lumen of the diverticulum. Nonetheless, accurate identification of the diverticulum is a difficult task and requires extensive experience.

NIR fluorescence with ICG has been used clinically for decades. The application of this method includes the identification of thoracic ducts, segmental borders, pulmonary nodules and bullous lesions [5]. To the best of our knowledge, there are few reports of this technique being used for diverticulectomy [6]. To ensure that the diverticulum neck was sufficiently coated with ICG, we inserted the lens of the endoscope into the diverticulum before injecting the ICG. In this case, we injected approximately 30 ml ICG (2.5 mg/ml) into the diverticulum through the endoscopic channel. Since the fluid needs to fill the endoscopic channel before it can enter the diverticulum, the amount of ICG can be slightly higher. Immediately after injection of ICG into the diverticulum, the diverticulum wall and neck were clearly visible under NIR fluorescence. Since NIR fluorescence with ICG can only penetrate a few millimeters of tissue, even if ICG flows into the esophagus, the esophageal wall near the diverticulum cannot be seen due to the muscle covering (Fig. 2C), which means that this technique has high specificity in identifying the diverticulum neck. Different from the use of endoscopic light, the diverticulum neck stained with ICG was visualized as a whole and was very stable under NIR fluorescence, so the surgeon can perform the excision of the diverticulum neck calmly.

Since the NIR fluorescence of ICG can only penetrate a few millimeters of tissue, a limitation of this technique is that it may only be used for false diverticula or true diverticula with thin muscular layers. The pathology of this case suggests that the diverticulum is a true diverticulum with a thin muscular layer. When the diverticulum has adhesions to the surrounding tissue, especially when the epiphrenic diverticulum can sometimes be adherent, this method may help to accurately identify the diverticulum neck. The specific application scope and method of this technique need further research.

We applied ICG and NIR fluorescence during diverticulectomy to better identify the diverticulum neck. This method is safe, simple to perform, and effective and has the potential to increase the efficiency of diverticulectomy.

## Data Availability

The data underlying this article will be shared upon reasonable request to the corresponding author.

## Abbreviations

MIS: Minimally invasive surgery
VATS: Video-assisted thoracoscopic surgery
ICG: Indocyanine green
NIR: Near-infrared
